# Efficient method to create integration-free, virus-free, *Myc* and *Lin28*-free human induced pluripotent stem cells from adherent cells

**DOI:** 10.4155/fsoa-2017-0028

**Published:** 2017-05-12

**Authors:** Anant Kamath, Sara Ternes, Stephen McGowan, Anthony English, Rama Mallampalli, Alan B Moy

**Affiliations:** 1 Cellular Engineering Technologies, Inc., 2500 Crosspark Rd, E110, Coralville, IA 52241, USA; 2 The John Paul II Medical Research Institute, Iowa City, IA, USA; 3 Department of Internal Medicine, Roy J & Lucille A Carver College of Medicine, University of Iowa, Iowa City, IA, USA; 4 Department of Biomedical Engineering, Western New England University College of Engineering, Springfield, MA, USA; 5 Division of Pulmonary, Allergy & Critical Care, University of Pittsburgh Medical Center Health System, Pittsburgh, PA, USA; 6 Department of Biomedical Engineering, University of Iowa, Iowa City, IA, USA

**Keywords:** episomal, IPS cell, reprogramming

## Abstract

**Aim:**

Nonviral induced pluripotent stem cell (IPSC) reprogramming is not efficient without the oncogenes, *Myc* and *Lin28*. We describe a robust *Myc* and *Lin28*-free IPSC reprogramming approach using reprogramming molecules.

**Methods:**

IPSC colony formation was compared in the presence and absence of *Myc* and *Lin28* by the mixture of reprogramming molecules and episomal vectors.

**Results:**

While more colonies were observed in cultures transfected with the aforementioned oncogenes, the *Myc* and *Lin28*-free method achieved the same reprogramming efficiency as reports that used these oncogenes. Further, all colonies were fully reprogrammed based on expression of SSEA4, even in the absence of *Myc* and *Lin28*.

**Conclusion:**

This approach satisfies an important regulatory pathway for developing IPSC cell therapies with lower clinical risk.

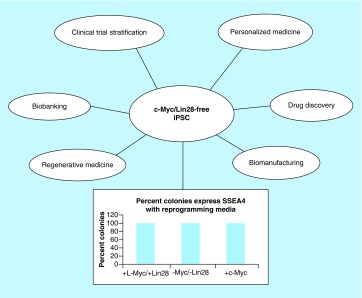

## Lay abstract

Induced pluripotent stem cells (IPSCs) avoid the ethical controversy of embryonic stem cells (ESCs), but carry the same neoplastic risk of embryonic stem cell because of two deployed oncogenes (*c-Myc* and *Lin28*). Pioneering work by Dr. Yamanaka showed that IPSC could be uncoupled from the neoplastic propensity by eliminating *c-Myc*. However, an oncogene-free and virus-free method has not been available. This report describes the first virus-free and oncogene-free IPSC method that efficiently produces pluripotent stem cells from adherent cells by the addition of reprogramming molecules. This approach satisfies an important regulatory pathway for developing safer future IPSC cell therapies.

Chronic disease from degenerative organ dysfunction accounts for 86% of our nation’s healthcare cost [1]. While organ transplantation is a definitive treatment for several end-staged organ disorders, there is an inadequate supply of available organ donations [2,3]. Stem cell therapy represents a potential solution to fill the gap of limited organ donations at a decreased cost. Pluripotent stem cells may represent a viable alternative and cost-effective regenerative medicine solution for several chronic conditions such as macular degeneration, cardiopulmonary disease, cancer, CNS disorders, metabolic diseases, and chronic liver and kidney disorders. Although human embryonic stem cells (ESCs) represent the first described pluripotent stem cells [4], these cells pose specific shortcomings. Notwithstanding its ethical controversy, ESC exhibits a neoplastic propensity if terminally differentiated cell cultures contain any undifferentiated cells. Further, there is a risk of graft rejection from human leukocyte antigen mismatch between donor and recipient. Thus, there have been efforts to develop alternative pluripotent stem cells that lack such ethical controversy and could avoid the immunogenicity and tumorigenicity that is inherent in ESC.

Induced pluripotent stem cells (IPSCs) represent a noncontroversial source of pluripotent stem cells that could achieve these objectives. A critical advantage of IPSC is the immunological compatibility that exists with autologous cell therapy. Takahashi *et al.* were the first to report the dedifferentiation of somatic fibroblasts into pluripotent stem cells by retroviral gene delivery of *Oct3/4, Sox2, Klf4* and *c-Myc* [5,6]. Yu *et al.* also reported creating cultured IPSC from fetal and neonatal fibroblasts by retroviral delivery of *Oct4, Sox2, Nanog and Lin28* [7]. Both groups demonstrated that pluripotent stem cells had similar characteristics to those reported in human ESCs. Nakagawa *et al.* further observed that deletion of *c-Myc* from the reprogramming scheme still created pluripotent colonies but eliminated teratoma formation [8]. Yet, the authors reported a significantly lower reprogramming efficiency under this condition even when retroviral gene delivery was deployed. Nakagawa *et al.* published a follow-up report demonstrating that replacement of *c-Myc* with *l-Myc* eliminated the neoplastic effects associated with *c-Myc* [9]. However, these observations were again conducted with retroviral systems. While *l-Myc* does not promote teratoma formation in murine models in short-term experiments, *l-Myc* has been associated with several clinical malignancies [10–12]. Also, heterologous expression of *c-Myc* as described in this report led to a much lower fraction of fully reprogrammed colonies than those created from heterologous *l-Myc* expression [9]. Taken together, these data indicate that the oncogenes, *c-Myc* and *Lin28*, are the chief determinants of the neoplastic risk associated with current IPSC reprogramming methods.

Episomal reprogramming is an ideal method for creating clinical-grade, safer, nonviral and nonintegrating IPSC. Exogenous genes introduced through episomal vectors can be easily monitored through fluorescent tags such as red fluorescent protein (RFP) and their shut down can be easily detected. Unlike other methodologies, episomal vectors are only active, on average for 17–21 days, before reaching an undetectable level due to dilution and instability caused by cell division. However, episomal reprogramming has often been avoided since the efficiency has historically been very low compared with other nonintegrating methods. To compensate for the lower reprogramming efficiency, episomal constructs have utilized *c-Myc* or a combination of *l-Myc* and *Lin28* [13–15]. Yu *et al.* previously reported no colony formation using an episomal reprogramming strategy that delivered *Oct4, Sox2, c-Myc, Nanog, Lin28 and Klf4* [16]. However, the authors reported colony formation at an efficiency of approximately 0.0006% only after the addition of an *SV40 large T-antigen* gene. In contrast, Okita *et. al.* previously reported a similar quantitative reprogramming efficiency from episomal-derived IPSC colonies with p53 suppression combined with *l-Myc* and *Lin28* heterologous expression [13].

In this manuscript, we report a robust and cost-effective episomal IPSC reprogramming strategy using adherent cells. The method is based on a combinatorial approach of reprogramming molecules combined with a mixture of episomal vectors that create IPSC without the need for *l-Myc*, *c-Myc* and *Lin28*. The reprogramming approach provides an IPSC reprogramming method that is virus-free, *Myc* and *Lin28*-free. Also, the IPSC reprogramming and cell expansion media is xeno-free and feeder-free. In this report, we quantify and compare the IPSC reprogramming efficiency of our combinatorial reprogramming approach in the following groups: between adherent human skin fibroblasts transfected with *c-Myc, l-Myc/Lin28* and Myc/Lin28-free constructs in the presence and absence of reprogramming molecules. IPSC reprogramming efficiency was defined using two criteria: the number of colonies that were created per 100,000 of input cells; and the fraction of those colonies that are fully reprogrammed based on the expression of SSEA-4, a biomarker of pluripotency.

## Methods

### Cultured human foreskin fibroblasts

Cultured neonatal foreskin fibroblasts were isolated from discarded foreskin obtained by routine circumcisions through an Institutional Review Board approved informed consent. Isolated cultured cells were de-identified in accordance with Institutional Review Board procedures.

### Episomal vector production

**Figure 1.**
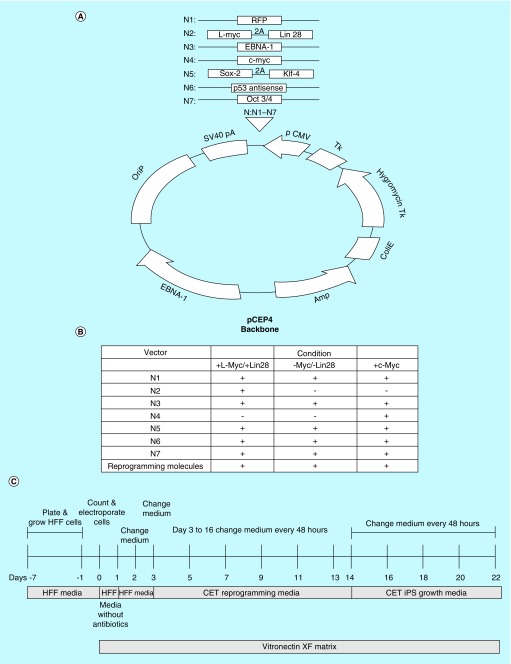
**Vector maps for the various episomal constructs.** **(A)** Generalized vector map of the episomal vector. Each vector is based on the pCEP-4 episomal backbone containing an EBV origin of replication (OriP), SV40 poly adenylation sequence, 2A cleavage sequence for tandem genes, a bacterial origin of replication and ampicillin/hygromycin resistance genes. Each vector either contains a single gene or tandem genes separated by a 2A cleavage sequence. See text for details. IPSC vector mixture of reprogramming molecules and episomal vectors for conditions; **(B)** A table includes the mixture of episomal vectors and reprogramming molecules that are used for IPSC reprogrammed with *c-Myc*; a combination of *l-Myc* and *Lin28*; and one free of *Myc* and *Lin28*. Time sequence of IPSC reprogramming and cell expansion; **(C)** Figure depicts the timeline sequence of IPSC reprogramming and cell expansion. IPSC: Induced pluripotent stem cell.

Each vector is based on the pCEP-4 episomal vector previously developed by ThermoFisher Scientific (MA, USA) (Figure 1A). It contains an Epstein–Barr virus (EBV) origin of replication, SV40 polyadenylation sequence, 2A cleavage sequence for tandem genes, a bacterial origin of replication and ampicillin/hygromycin resistance genes. Each vector either contains a single gene or tandem genes separated by a 2A cleavage sequence. In addition to the traditional Yamanaka factors (*Oct3/4, Sox2 and Klf4*), there are separate vectors containing *l-Myc* coupled with *Lin28* and separate episomal vectors that encodes for *p53* antisense and *c-Myc*. The system also contains a vector that encodes for RFP to monitor gene delivery and to detect silencing of exogenous reprogramming factors. Last, for each of the vectors to efficiently remain in the cell cytoplasm for only a short time frame, the plasmid vector encodes for Epstein–Barr Nuclear Antigen-1 (EBNA-1). Three separate IPSCs were created with cultured human foreskin fibroblasts (HFFs), which were reprogrammed with *c-Myc*; a combination of *l-Myc* and *Lin28*; and one free of *Myc* and *Lin28*. The specific vectors and reprogramming molecules used under each condition are depicted in a table (Figure 1B).

### IPSC reprogramming & colony expansion

A temporal sequence of the IPSC reprogramming and cell expansion process is depicted in Figure 1C. Prior to transfection, a 6-well dish was coated with Vitronectin-XF according to manufacturer’s directions (Primorigen, WI, USA). HFF cells were examined under a microscope to ensure logarithmic growth phase and 80% confluency. HFF cells were washed with 1× Dulbecco’s phosphate-buffered saline (ThermoFisher Scientific). HFF cells were then exposed to 0.25% Trypsin-EDTA (ThermoFisher Scientific) and incubated at 37°C for 4 min. When the cells were no longer adherent, an equal amount of 10% fetal bovine serum containing HFF growth media without antibiotics/antifungal was added. HFF cells were counted and the density was adjusted to 1 × 10^5^ cells/ml. HFF cells were spun to pellet at 200 × *g* for 5 min. Each cell pellet, containing 1 × 10^5^ cells/ml, was resuspended in 100 μl of Neon Electroporation Buffer R (ThermoFisher Scientific). A total of 3.5 μg of DNA of the episomal reprogramming mix (Cellular Engineering Technologies, IA, USA) was added to each tube and mixed gently. A Neon Electroporation Tip-100 was used to introduce the cells to the DNA. Using Buffer E2 for the chamber buffer, the cells were electroporated at 1650 V for 10 ms for 3 cycles. Immediately after electroporation, the cells were placed in HFF growth media containing no antibiotics/antifungals on the previously coated 6-well dish for the first 24 h.

After 24 h, the growth media were withdrawn and replaced with the IPSC reprogramming media (Cellular Engineering Technologies) containing antibiotics/antifungals. Reprogramming media consisted of 1× DMEM/F12 with HEPES (ThermoFisher Scientific), 1× N-2 Supplement (ThermoFisher Scientific), 1× B-27 Supplement (ThermoFisher Scientific), 1× MEM Non-Essential Amino Acids (ThermoFisher Scientific) 1× Glutamax (ThermoFisher Scientific) and 1× β-mercaptoethanol (ThermoFisher Scientific). A reprogramming mixture was added which contained Human Recombinant FGF-2 (Peprotech, NJ, USA), sodium butyrate (Reagents Direct, CA, USA), ascorbic acid (Sigma-Aldrich, MO, USA), A83–0–1 (Reagents Direct) and PS48 (Reagents Direct). To evaluate successful transfection, cells were examined under a microscope to detect RFP fluorescence within the first 48 h. Cells were fed with fresh IPSC reprogramming media every 48 h through day 14 of the reprogramming process. From day 15 onward, a full media replacement was performed every 24 h with a defined xeno-free, IPSC growth media (Cellular Engineering Technologies). Mature IPS colonies were observed starting around day 17 post electroporation, which displayed sharp and distinct borders. The identity of the IPS colonies was confirmed with positive probes for various IPSC markers including SSEA-4 live stain (ThermoFisher Scientific) and alkaline phosphatase (Stemgent, MA, USA).

### Statistical analysis

IPSC reprogramming efficiency (expressed as a percentage) was defined by the following formula: number of colonies counted per 100,000 input cells × 100. Data are reported as means ± SE. Comparisons between more than two groups were made with analysis of variance. Individual group comparisons were done with Tukey’s honestly significant difference test for post hoc comparison of means. Differences were considered significant at the p ≤ 0.05 level.

## Results

We generated several episomal vector constructs which are illustrated in Figure 1A. There were seven separate vectors, which encode for a unique single reprogramming gene or tandem reprogramming genes separated by a 2A cleavage sequence. Cultured cells were reprogrammed under three separate conditions. Each condition contained a mixture of vectors that contain genes that encode for Oct3/4, Sox2, Klf-4, EBNA-1, p53 antisense and RFP proteins. One group of cultured cells was reprogrammed with an additional vector that contained *l-Myc* and *Lin28* separated by a 2A cleavage sequence. A separate group of cultured cells was reprogrammed with an additional vector that encoded for the gene that expressed c-Myc. Last, one group of cultured cells was reprogrammed without *Myc* and *Lin28*. Figure 1B depicts a table that summarizes the different combination of transcriptional factors and reprogramming molecules. An input of 100,000 cultured HFF cells was transfected by the corresponding vector mixture for each condition. All the cultured conditions were electroporated and sequentially exposed to an IPSC reprogramming media followed by IPSC growth media in accordance with the timeline illustrated in Figure 1C. Cultured cells were electroporated in the presence of a reprogramming media containing reprogramming molecules and grown in media for 14 days. Cultured cells were then switched to a xeno-free, feeder-free, growth media for an additional 7 days. By day 22 the number of colonies were counted and stained with alkaline phosphatase or SSEA4 live stain.

**Figure 2.**
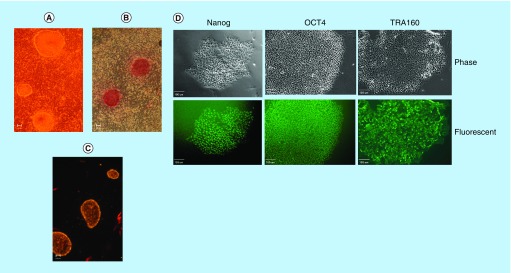
**Montage of cultured human foreskin fibroblast reprogrammed into induced pluripotent stem cell with episomal vectors free of *Myc* and *Lin28*, and induced pluripotent stem cell reprogramming molecules.** Images were captured at day 14 of the IPSC reprogramming process. **(A)** Typical IPSC colonies depicted by phase contrast microscopy. **(B)** Representative IPSC colonies stain for alkaline phosphatase. **(C)** Representative IPSC colonies exhibit pluripotency by immunofluorescent live stain for SSEA4. Each figure is representative of four separate experiments. Scale bar represents 100 μm. **(D)** Montage of representative IPSC colonies depicted by phase microscopy with corresponding pluripotent fluorescent biomarker of Nanog, Oct4 and TRA160 of the same colony. Scale bar represents 100 μm. IPSC: Induced pluripotent stem cell.

By day 14, IPSC colonies were typically formed as shown under phase microscopy (Figure 2A). The colony exhibits the typical flat shape and refractile border. IPSC colonies also stain positive for alkaline phosphatase (Figure 2B). Colonies also expressed SSEA4 (Figure 2C), which confirm that the reprogramming process resulted in fully reprogrammed cells. Additionally, representative colonies depicted by phase microscopy and other pluripotent biomarkers (Nanog, Oct4 and TRA160) were observed within the same corresponding colony (Figure 2D).

**Figure 3.**
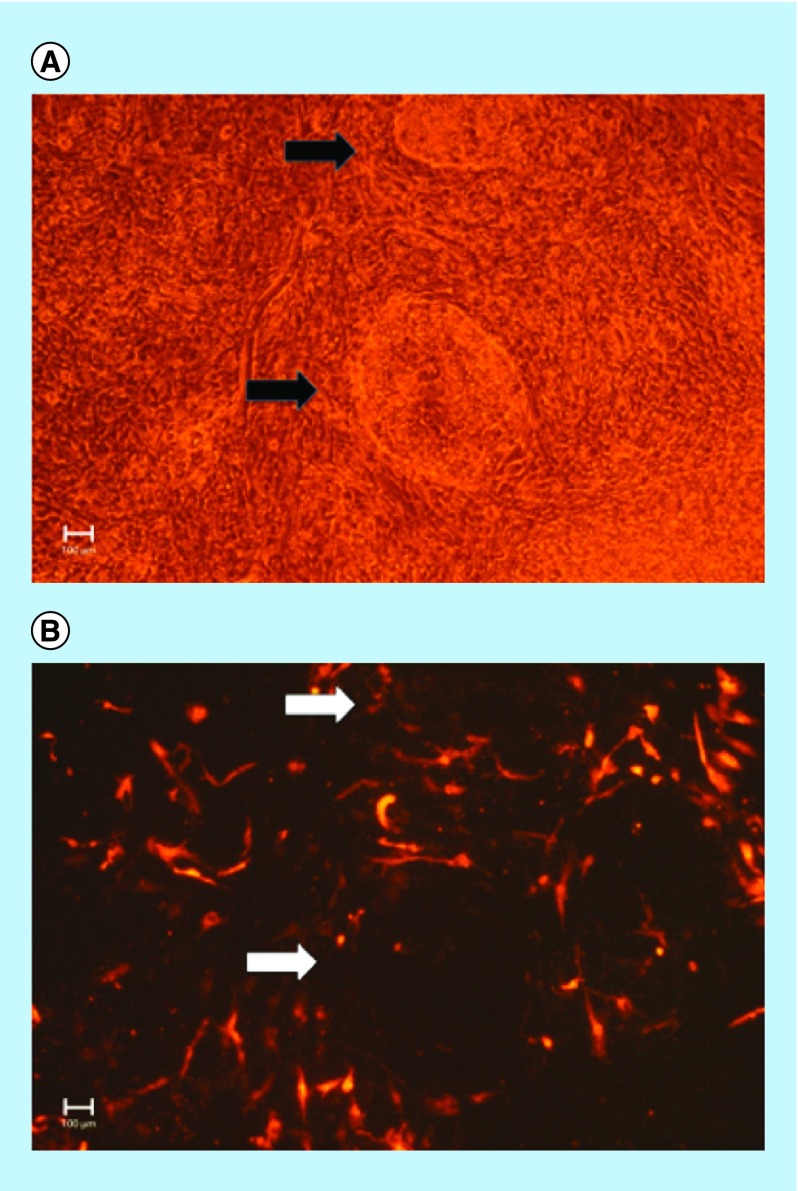
**Ectopic episomal vectors are shut down in cultured induced pluripotent stem cell colonies within 2 weeks.** Reprogrammed colonies formed through a combinatorial method of reprogramming molecules and a mixture of episomal vectors free of *Myc* and *Lin28* are shut down. The figure shows the expression of RFP in culture IPSC colonies at day 17. **(A)** Black arrows point to IPSC colonies captured under phase microscopy. **(B)** White arrows point to the corresponding RFP signal in the same colonies. As shown there is a complete loss of expression of RFP in the IPSC colonies demonstrating that episomal vectors are shut down within 2 weeks. The figure is representative of four separate experiments. Scale bar represents 100 μm. IPSC: Induced pluripotent stem cell; RFP: Red fluorescent protein.

To confirm that the episomal vectors were silenced within a short time frame, immunofluorescent experiments were conducted that examined the expression of RFP in the IPSC colonies (Figure 3). As shown in the Figure 3A, there are two IPSC colonies that are highlighted under phase microscopy at day 17. The corresponding fluorescent images (Figure 3B) show that the IPSC colonies no longer expressed RFP, which indicate that the genes encoded by the episomal vectors had shutdown.

**Figure 4.**
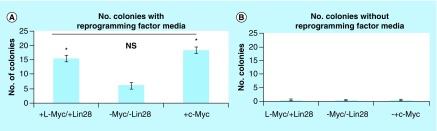
**The number of induced pluripotent stem cell colonies created in the presence and absence of induced pluripotent stem cell reprogramming molecules.** **(A)** Figure illustrates the number of colonies generated between the different vector constructs among cultured cells reprogrammed in the presence of reprogramming molecules. Data are reported as the mean (±SE) number of colonies observed for cultured HFF reprogrammed with *l-Myc/Lin28*, *c-Myc* and in the absence of both oncogenes. Each test condition used 100,000 input cells. Each group represents a sample size of four. **(B)** Illustrates the number of colonies generated between the different vector constructs among cultured cells reprogrammed in the absence of reprogramming molecules. Data are reported as the mean (±SE) number of colonies observed for cultured HFF reprogrammed with *l-Myc/Lin28*, *c-Myc* and in the absence of both oncogenes. Each group represented a sample size of four. Data labeled with * show a statistical significant difference (p < 0.05) between cultured cells treated with Myc and Lin28 and those cells treated without *Myc* and *Lin28*. NS denotes no significant difference. HFF: Human foreskin fibroblast; SE: Standard error.

Next, the number of colonies generated between the different vector constructs was compared among cultured cells reprogrammed in the presence and absence of reprogramming molecules (Figure 4). As shown each construct resulted in several IPSC colonies. There were enough colonies that were observed in cells transfected without *l-Myc, Lin28* and *c-Myc* (Figure 4A). Yet, there was a statistically greater number of colonies observed in cultured cells transfected when either *l-Myc* combined with *Lin28* or when *c-Myc* alone was added. There was no statistically significant difference in the number of colonies formed between cells transfected with *l-Myc* combined with *Lin28* and those transfected with *c-Myc*. When the same vector mixtures were transfected in cultured cells in the absence of reprogramming molecules, there was 0–1 colony detected (Figure 4B) irrespective whether in the presence or absence of *Myc*-dependent and *Lin28* transcriptional factors. Clearly, there was a higher number of IPSC colonies when cultured cells were treated with the reprogramming molecules. We have also performed experiments where we omitted any one of the four reprogramming factors. There were essentially no colonies that formed under those conditions (data not shown). These observations were consistent regardless if *c-Myc* or *Lin28* was included or not. Thus, all four reprogramming factors were necessary to efficiently form IPSC using this episomal reprogramming system.

**Figure 5.**
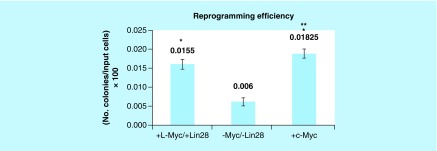
**Impact of induced pluripotent stem cell reprogramming molecules in the presence and absence of *Myc* and *Lin28*.** Reprogramming efficiency is expressed as the percentage of colonies counted per 100,000 of input cells × 100. Data are reported as the mean (±SE). Each group represents a sample size of four replicates. Data labeled with * highlight a statistical significant difference (p < 0.05) between cultured cells treated with *Myc* and *Lin28* and those cells treated without *Myc* and *Lin28*. ** denotes a significant statistical difference (p < 0.05) between cells transfected with *c-Myc* and *l-Myc/Lin28*. SE: Standard error.

When expressed as the percentage of colonies counted per 100,000 of input cells, there was a parallel significant difference in the reprogramming efficiency between cells transfected in the presence and absence of *Myc*-dependent and *Lin28* transcriptional factors (Figure 5). Interestingly, cultured cells transfected with *c-Myc* resulted in a statistically higher reprogramming efficiency than cultured cells transfected with *l-Myc* combined with *Lin28*.

**Figure 6.**
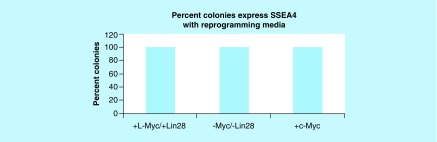
Induced pluripotent stem cell **reprogramming with reprogramming molecules exhibit the same percentage of pluripotent colonies in the presence and absence of *Myc/Lin28*.** Figure depicts the percentage of colonies that express SSEA4 among cultured human foreskin fibroblast exposed to *l-Myc/Lin28*, *c-Myc* and the absence of both oncogene groups. Data are reported as the mean (standard error = 0). All colonies stained positive for SSEA4. Each group represents a sample size of four replicates.

Next, reprogramming efficiency was further quantified by measuring the fraction of colonies that expressed SSEA4 when exposed to reprogramming molecules. As shown in Figure 6, all the colonies were fully reprogrammed irrespective of whether the cells were transfected in the presence or absence of *Myc*-dependent and *Lin28* transcriptional factors (standard error = 0). Taken together, the data demonstrate that our approach of combining reprogramming molecules with mixtures of episomal vectors that lacked *Myc* and *Lin28* created enough IPSC colonies in which all were fully reprogrammed.

## Discussion

We report an IPSC reprogramming method, which uses a combinatorial approach of reprogramming molecule enhancers and a mixture of episomal vectors that are free of *Myc* and *Lin28*. The combinatorial approach produced enough fully reprogramed IPSC colonies. Although the transfection of *l-Myc* and *c-Myc* mediated an anticipated greater number of colonies than in the absence of these genes, all transfection groups, including those treated in the absence of *c-Myc* and *l-Myc* expression vectors, yielded colonies in which 100% were fully reprogrammed. Thus, our reprogramming method showed that colonies were fully reprogrammed irrespective of whether oncogene transcriptional factors were used or not. In contrast, we observed at most one colony formation for 100,000 input cells in the absence of reprogramming molecules regardless of whether cells were transfected in the presence or absence of *Myc* and *Lin28* transcriptional factors, which is in agreement with other reports [16]. Since cultured cells were treated with an excess of exogenous DNA, it is likely that more colonies could be formed in the absence of reprogramming molecules if a higher number of input cells were used. Thus, our combination of reprogramming molecules served as a catalyst for successfully reprogramming adherent target cells without the need to use *Lin28* and *Myc*-dependent transcriptional factors.

The reprogramming efficiency with our *Myc* and *Lin28*-free combinatorial approach (0.006%) exceeds that of other reports that used episomal reprogramming vectors containing *Myc* and *Lin28*. Yu *et al.* previously reported no colony formation using an episomal reprogramming strategy that delivered *Oct4*, *Sox2*, *Nanog*, *Lin28*, *c-Myc* and *Klf4* [16]. However, the authors reported an efficiency of approximately 0.0006% only after the addition of an *SV40 large T* gene using 1 million input cells. Further, our reprogramming efficiency exceeds that reported by Okita *et al.* in which the authors used a combination of *l-Myc* and *Lin28* in their reprogramming scheme [13].

The reprogramming efficiency of *l-Myc* and *c-Myc* IPSC reprogramming in our system was twice greater than that of the oncogene-free vector combination. We also observed that our reprogramming molecule enhancers exhibited a greater fraction of colonies that achieved a fully reprogrammed state than prior reports [9]. In contrast to prior reports we observed a greater reprogramming efficiency with *c-Myc* than with *l-Myc* [13]. The reason for these differences remains unclear.

The reprogramming molecule mixture enhanced IPSC reprogramming by activating several signaling pathways. These signaling pathways were promoted by using sodium butyrate as a histone deactylase inhibitor; ascorbic acid to mitigate oxidative stress and increase cellular division; A83–0–1 as an Alk-5 inhibitor; and PS48 as a phosphoinositide-dependent protein kinase-1 inhibitor, which facilitates a conversion from mitochondrial oxidation to glycolysis. Unlike single agents which have been used by other investigators [17], we sought to develop a reprogramming molecule mixture that capitulated the temporal embryological events of the target cells from introduction of reprogramming factors to the shutdown of exogenous genes [18]. This approach resulted in completely reprogrammed IPSC. In doing so, the main considerations were the reduction of apoptosis in reprogramming cells [19], erasing the epigenetic memory of target cells [18], maintaining fully reprogrammed or ESC-like cells [18] and addressing the metabolic switch from aerobic respiration to glycolytic dependence seen in ESC and IPSC [20]. By formulating this combination of reprogramming molecules, we could develop a reagent that addressed each of these requirements. The reprogramming molecule mixture compensates for the low efficiency or lack of reprogramming other investigators have reported for episomal IPSC reprogramming methodologies. Moreover, it results in fully reprogrammed IPSC that are integration-free with a complete shutdown in the expression of exogenous genes, which is a critical factor in reducing the neoplastic risk.

The pursuit of virus-free IPSC reprogramming methods for cell therapy is needed to eliminate important safety risks as an IPSC-specific cellular therapy. First, viral reprogramming poses an obvious infectious risk. Second, retroviral gene delivery results in random genomic integration of exogenous DNA, which increase the oncogenic risk by random silencing of tumor suppressor genes [21]. Thus, footprint-free or nongenomic integrating and nonviral methods are preferred to produce clinical grade IPSC. Nonviral reprogramming methods such as piggyback [22], DNA minicircles [23] and microRNA [24] are extremely inefficient. Thus, developing a robust and straightforward IPSC reprogramming method is required to convert geriatric target cells into pluripotent stem cells [25–29]. This is the age group most likely affected from chronic diseases; most often excluded from organ transplantation; and most in need of regenerative medicine therapy.

There are currently four principal nonintegrating IPSC reprogramming methods that are commonly used: self-replicating or replicon RNA [30], mRNA reprogramming [31–33], Sendai virus reprogramming [34–37] and episomal plasmid reprogramming [13,16]. Self-replicating or replicon RNA relies on the Venezuelan equine encephalitis virus positive sense, single-stranded RNA backbone and its ability to mimic cellular mRNA without having a DNA intermediate. While the lack of a DNA intermediate obviates integration hazards, it is unclear how usage of a virus backbone influences host immunity in IPSC that are created downstream. It is also known that to get an appreciable level of reprogramming with self-replicating RNA, transfection with co-agents that suppress immune response are necessary [30]. Moreover, PCR studies have shown the retained expression of viral RNA components for at least four passages downstream, making IPS colony selection, especially for therapeutics a lengthy process. mRNA-based reprogramming [31–33] requires repeated daily transfections for up to 17 days and is laborious and expensive. Also, the method must contend with interferon production in transfected cells, which impacts reprogramming efficiency and which could present downstream immunological concerns. While Sendai virus reprogramming is a popular and robust IPSC reprogramming method, a far greater number of cell divisions are required to create an established cell line free of contaminating viral proteins [34]. Ultimately, IPSC colonies would have to be carefully screened for viral proteins before chosen for a cell therapy. Thus, Sendai virus reprogramming is not an efficient method for developing cell therapies.

The use of an episomal expression system has the advantage over other reprogramming methods by silencing ectopic transcriptional factors within a short time window. We observed that ectopic expression of transcriptional factors was eliminated within 2 weeks based on the absence of expression of RFP in IPSC colonies. The temporal loss of EBV vectors in our hands is consistent with other reports on the cell cycle of EBV vectors [38]. The ultimate loss of ectopic expression of reprogramming factors ensures that cells will unlikely exhibit dysregulated cell growth.

Our reprogramming method provides patient and disease-specific IPSC for drug discovery and personalized medicine applications with lower risk of oncogenic perturbations due to *Lin28* and *Myc*. More importantly, the technology creates a regulatory pathway for large scale manufacturing of IPSC-derivative cell therapies for a variety of chronic diseases in which the manufacturing process should reduce infectious and oncogenic risk. The reprogramming method paves a pathway for autologous and allogeneic cell therapy that satisfies regulatory requirements. The reprogramming method for converting adherent cells into IPSC is cost effective, efficient and provides an extremely high yield of pluripotent conversion and purity.

## Conclusion

The combination of episomal vectors and mixture of reprogramming molecules produces an efficient scheme of creating IPSC without the need to introduce *Myc* and *Lin28*.

## Future perspective

It is anticipated that virus-free and oncogene-free IPSC will touch many areas of biotechnology and healthcare. First, the technology will advance autologous and allogeneic cell therapy for unmet medical needs and replace underperforming treatments for chronic diseases with regenerative medicine solutions. Second, the technology should provide the next generation of diagnostic tools in personalized medicine and biobanking. Third, the technology should reduce the high failure rate in drug development with predictive preclinical drug screening by eliminating viral and oncogenic factors that could skew pharmacological responses. Fourth, the failure rate of clinical trials could be dramatically improved by incorporating such technology in patient stratification and recruitment. Last, the technology has the potential for developing improved and ethically noncontroversial human cell lines for manufacturing human biologics, which currently rely on established cell lines.

**Summary points**

- Mixture of induced pluripotent stem cell (IPSC) reprogramming molecules efficiently reprograms adherent fibroblasts through an episomal expression system without a need for *Myc* and *Lin28*.
- A total of 100% of reprogrammed colonies expressed SSEA4 in the presence of reprogramming molecules.
- The efficiency of *Myc* and *Lin28*-free episomal IPSC reprogramming in the presence of reprogramming molecules exceeds that of prior reports of IPSC episomal reprogramming efficiency that required *Myc* and *Lin28*.
- The IPSC reprogramming method is viral-free, integration-free, Matrigel-free, oncogene-free (*Myc* and *Lin28*-free) and feeder-free, which meets regulatory requirements for developing derivative IPSC therapy with potentially lower infectious and neoplastic risk.

## References

[1] Gerteis J, Izrael D, Deitz D (2014). Multiple Chronic Conditions Chartbook. *Agency for Healthcare Research and Quality*.

[2] Khan AM, Green RS, Lytrivi ID, Sahulee R (2016). Donor predictors of allograft utilization for pediatric heart transplantation. *Transpl. Int.*.

[3] Thiessen C, Kulkarni S, Reese PP, Gordon EJ (2016). A call for research on individuals who opt out of living kidney donation: challenges and opportunities. *Transplantation*.

[4] Thomson JA, Itskovitz-Eldor J, Shapiro SS (1998). Embryonic stem cell lines derived from human blastocysts. *Science*.

[5] Takahashi K, Tanabe K, Ohnuki M (2007). Induction of pluripotent stem cells from adult human fibroblasts by defined factors. *Cell*.

[6] Takahashi K, Yamanaka S (2006). Induction of pluripotent stem cells from mouse embryonic and adult fibroblast cultures by defined factors. *Cell*.

[7] Yu J, Vodyanik MA, Smuga-Otto K (2007). Induced pluripotent stem cell lines derived from human somatic cells. *Science*.

[8] Nakagawa M, Koyanagi M, Tanabe K (2008). Generation of induced pluripotent stem cells without *Myc* from mouse and human fibroblasts. *Nat. Biotechnol.*.

[9] Nakagawa M, Takizawa N, Narita M, Ichisaka T, Yamanaka S (2010). Promotion of direct reprogramming by transformation-deficient *Myc*. *Proc. Natl Acad. Sci. USA*.

[10] Ikegaki N, Minna J, Kennett RH (1989). The human *L-Myc* gene is expressed as two forms of protein in small cell lung carcinoma cell lines: detection by monoclonal antibodies specific to two myc homology box sequences. *EMBO J.*.

[11] Bektas-Kayhan K, Unur M, Yaylim-Eraltan I (2009). Role of *l-Myc* polymorphism in oral squamous cell carcinoma in Turkey. *Anticancer Res.*.

[12] Yaylim-Eraltan I, Bozkurt N, Ergen A (2008). *L-Myc* gene polymorphism and risk of thyroid cancer. *Exp. Oncol.*.

[13] Okita K, Matsumura Y, Sato Y (2011). A more efficient method to generate integration-free human iPS cells. *Nat. Methods*.

[14] Yin X, Li Y, Li J (2016). Generation and periodontal differentiation of human gingival fibroblasts-derived integration-free induced pluripotent stem cells. *Biochem. Biophys. Res. Commun.*.

[15] Zhao T, Zhang ZN, Rong Z, Xu Y (2011). Immunogenicity of induced pluripotent stem cells. *Nature*.

[16] Yu J, Hu K, Smuga-Otto K (2009). Human induced pluripotent stem cells free of vector and transgene sequences. *Science*.

[17] Ichida JK, Blanchard J, Lam K (2009). A small-molecule inhibitor of TGF-Beta signaling replaces *Sox2* in reprogramming by inducing nanog. *Cell Stem Cell*.

[18] Medvedev SP, Shevchenko AI, Zakian SM (2010). Induced pluripotent stem cells: problems and advantages when applying them in regenerative medicine. *Acta Naturae*.

[19] Tapia N, Scholer HR (2010). p53 connects tumorigenesis and reprogramming to pluripotency. *J. Exp. Med.*.

[20] Panopoulos AD, Yanes O, Ruiz S (2012). The metabolome of induced pluripotent stem cells reveals metabolic changes occurring in somatic cell reprogramming. *Cell Res.*.

[21] Suzuki T, Minehata K, Akagi K, Jenkins NA, Copeland NG (2006). Tumor suppressor gene identification using retroviral insertional mutagenesis in Blm-deficient mice. *EMBO J.*.

[22] Woltjen K, Michael IP, Mohseni P (2009). piggyBac transposition reprograms fibroblasts to induced pluripotent stem cells. *Nature*.

[23] Jia F, Wilson KD, Sun N (2010). A nonviral minicircle vector for deriving human iPS cells. *Nat. Methods*.

[24] Anokye-Danso F, Trivedi CM, Juhr D (2011). Highly efficient miRNA-mediated reprogramming of mouse and human somatic cells to pluripotency. *Cell Stem Cell*.

[25] Yagi T, Kosakai A, Ito D (2012). Establishment of induced pluripotent stem cells from centenarians for neurodegenerative disease research. *PLoS ONE*.

[26] Banito A, Rashid ST, Acosta JC (2009). Senescence impairs successful reprogramming to pluripotent stem cells. *Genes Dev.*.

[27] Kawamura T, Suzuki J, Wang YV (2009). Linking the p53 tumour suppressor pathway to somatic cell reprogramming. *Nature*.

[28] Li H, Collado M, Villasante A (2009). The *Ink4/Arf* locus is a barrier for iPS cell reprogramming. *Nature*.

[29] Lapasset L, Milhavet O, Prieur A (2011). Rejuvenating senescent and centenarian human cells by reprogramming through the pluripotent state. *Genes Dev.*.

[30] Yoshioka N, Gros E, Li HR (2013). Efficient generation of human iPSCs by a synthetic self-replicative RNA. *Cell Stem Cell*.

[31] Rosa A, Brivanlou AH (2010). Synthetic mRNAs: powerful tools for reprogramming and differentiation of human cells. *Cell Stem Cell*.

[32] Warren L, Manos PD, Ahfeldt T (2010). Highly efficient reprogramming to pluripotency and directed differentiation of human cells with synthetic modified mRNA. *Cell Stem Cell*.

[33] Mandal PK, Rossi DJ (2013). Reprogramming human fibroblasts to pluripotency using modified mRNA. *Nat. Protoc.*.

[34] Fujie Y, Fusaki N, Katayama T (2014). New type of Sendai virus vector provides transgene-free iPS cells derived from chimpanzee blood. *PLoS ONE*.

[35] Isono K, Jono H, Ohya Y (2014). Generation of familial amyloidotic polyneuropathy-specific induced pluripotent stem cells. *Stem Cell Res.*.

[36] Kawagoe S, Higuchi T, Otaka M (2013). Morphological features of iPS cells generated from Fabry disease skin fibroblasts using Sendai virus vector (SeVdp). *Mol. Genet. Metab.*.

[37] Yang W, Mills JA, Sullivan S, Liu Y, French DL, Gadue P (2012). iPSC reprogramming from human peripheral blood using Sendai virus mediated gene transfer. *StemBook.*.

[38] Nanbo A, Sugden A, Sugden B (2007). The coupling of synthesis and partitioning of EBV’s plasmid replicon is revealed in live cells. *EMBO J.*.

